# From Bad to Good: Fitness Reversals and the Ascent of Deleterious Mutations

**DOI:** 10.1371/journal.pcbi.0020141

**Published:** 2006-10-20

**Authors:** Matthew C Cowperthwaite, J. J Bull, Lauren Ancel Meyers

**Affiliations:** 1 The Institute for Cellular and Molecular Biology, The University of Texas at Austin, Austin, Texas, United States of America; 2 Section of Integrative Biology, The University of Texas at Austin, Austin, Texas, United States of America; 3 Santa Fe Institute, Santa Fe, New Mexico, United States of America; University of Washington, United States of America

## Abstract

Deleterious mutations are considered a major impediment to adaptation, and there are straightforward expectations for the rate at which they accumulate as a function of population size and mutation rate. In a simulation model of an evolving population of asexually replicating RNA molecules, initially deleterious mutations accumulated at rates nearly equal to that of initially beneficial mutations, *without* impeding evolutionary progress. As the mutation rate was increased within a moderate range, deleterious mutation accumulation and mean fitness improvement both increased. The fixation rates were higher than predicted by many population-genetic models. This seemingly paradoxical result was resolved in part by the observation that, during the time to fixation, the selection coefficient (*s*) of initially deleterious mutations reversed to confer a selective advantage. Significantly, more than half of the fixations of initially deleterious mutations involved fitness reversals. These fitness reversals had a substantial effect on the total fitness of the genome and thus contributed to its success in the population. Despite the relative importance of fitness reversals, however, the probabilities of fixation for both initially beneficial and initially deleterious mutations were exceedingly small (on the order of 10^−5^ of all mutations).

## Synopsis

Mutations are the fuel of natural selection. It is widely believed that most mutations are deleterious, that is, they harm the organisms in which they occur. Thus, biologists would like to understand how deleterious mutations impact evolution. Most of the theoretical work on this problem makes an important assumption: mutations that start bad stay bad. It may be possible, however, for an initially bad mutation to become good (beneficial) by interacting with subsequent mutations. In this study, Cowperthwaite, Bull, and Meyers show that such “fitness reversals” are surprisingly common and can lead to the fixation of initially deleterious mutations. Perhaps mutations that undergo such changes serve as stepping stones for greater evolutionary progress.

## Introduction

Modern evolutionary theory recognizes that deleterious mutations may reduce fitness and retard adaptation [1–5]. Accumulation of deleterious mutations is expected to affect the rate and course of many biological processes such as sexual selection, development of cancer, and senescence [6]. The theoretical work underlying these predictions makes an important assumption: the fitness effect of a deleterious mutation is constant until the mutation disappears or fixes.

In the standard infinite population experiencing a combination of natural selection and random mutation, deleterious mutations should not fix, but accumulate to a level perfectly balanced by mutation and selection. Some processes can lead to deleterious mutations fixing in infinite populations, however. For example, in Eigen's quasispecies model, high rates of mutation can overwhelm selection and shift the mutation–selection balance such that deleterious mutations accumulate to exceedingly high levels [7,8]. In finite populations, several processes may also allow deleterious mutation fixation [9]. The best studied of these is random genetic drift—the stochastic fixation of deleterious mutations in relatively small populations. Additionally, if recombination is rare and the population size is finite, then deleterious mutations can hitchhike to fixation with independently acting beneficial mutations [10,11].

The fixation of deleterious mutations certainly reduces the fitness of populations. It may be possible, however, for the fitness effect of an initially deleterious mutation to change over time. In particular, compensatory mutations may evolve that reduce the negative impact of deleterious mutations or, in extreme cases, the resulting fitness may be even higher than the fitness of the ancestor in which the deleterious mutation arose [12]. Such compensatory mutations may appear before (or after) the deleterious mutation has fixed. Metaphorically speaking, while fixed deleterious mutations are generally expected to be bad, they may be stepping stones to distant adaptive peaks.

Evolutionary geneticists have long considered mutations that ameliorate or compensate for the deleterious effect of a prior mutation. The literature on this subject, however, focuses almost exclusively on compensatory mutations occurring after the fixation of the initial deleterious mutation, and therefore does not address the likelihood that the initial mutation will fix in the first place [13–15]. One possible explanation for this emphasis is convenience—both mathematical and experimental. To greatly simplify the evolutionary dynamics, population-genetic models of adaptation typically assume that selection is much stronger than mutation: strong selection, weak mutation (SSWM). Under this assumption, a deleterious mutation will disappear or fix before secondary mutations arise in the genome and thus the fitness effect of a deleterious mutation remains unchanged throughout its evolutionary trajectory to either fixation or loss [16].

If the mutation rate is relatively large, however, additional mutations may arise in the genome carrying the initially deleterious mutation before it fixes or is lost. Such secondary mutations change the genetic background and thus potentially change the fitness effect of the initial deleterious mutation. The background selection literature has frequently considered the scenario in which a good mutation is driven to extinction by bad mutations (see [11,17] and references therein). Our interest, however, is in a process involving epistasis between mutations that results in amelioration of the deleterious effect and, ultimately, the fixation of an initially bad mutation. Although this process seems to have been largely ignored in the population genetics literature, one notable study by Kimura shows that mutations at two linked alleles, which are singly deleterious but jointly neutral, can both fix relatively rapidly even in large populations [18]. There has also been recent interest in the ability of populations to escape from local optima via less fit intermediate genotypes (see [12] and references therein).

Here we use a computational model of asexually replicating RNA molecules to study the fixation of deleterious mutations. We first observe that initially deleterious mutations fix at a far greater rate than expected for an evolving asexual population and that some populations achieve high mean fitness despite rapidly accumulating deleterious mutations. We then reconcile this paradox by systematically characterizing the processes leading to fixation, which include random genetic drift, hitchhiking upon independently acting beneficial mutations, and fitness-effect reversals upon secondary (compensatory) mutations.

## Materials and Methods

### Simulation Model

We used a computational simulation of a population of replicating and evolving RNA molecules. Similar simulation models have been extensively used in previous studies of evolutionary dynamics [19–24]. The program, RNAvolver (available from MCC upon request), was designed to make straightforward comparisons to existing theory by simulating a stochastic, discrete-generation, asexually replicating population with a fixed size. The fitness function is based on the folding of RNA sequences into secondary structures [22,24,25]. The fitness effect of a mutation thus stems from a biologically explicit model of molecular structure and is not simply selected from a probability distribution of mutational effects, as in simpler evolutionary models [26].

In our model, the genotype of each member of the population is the primary RNA sequence of *L* = 76 nucleotides, which is similar in size to a typical tRNA molecule. The focal phenotype is RNA secondary structure (“shape,” informally), which provides the scaffold for functional tertiary structure and has been highly conserved during evolution [27]. In the simulation program, the “fitness” of each genotype is a function of its repertoire of probable secondary structures, which we predict using thermodynamic minimization [28–30]. The folding algorithm is relatively accurate for shorter molecules, but is not able to model pseudoknots (a common tertiary structure motif) and other noncanonical interactions [29,31,32].

Fitness depends on both similarity to a reference shape (the “target,” *t*) and thermodynamic stability, which is believed to impose a selective constraint on both naturally and artificially evolved RNA molecules [33]. To assign fitness to a molecule, we first predict the *ensemble* of lowest free energy shapes (all shapes within 3 kcal/mol of the groundstate) using the ViennaRNA-1.5 package [29,30] and then measure the structural difference between each shape (*σ*) in the ensemble and the target structure *t*. The selective value of a shape *σ* is given by

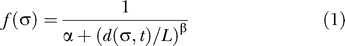
where *α* = 0.01 and *β* = 1 are scaling constants, *d*(*σ*,*t*) is the Hamming distance between *σ* and the target shape, and *L* = 76 is the length of the sequence. To determine the Hamming distance between two shapes, we measured the number of positions at which the parenthesized representations (e.g. ((((....)))), where matching parentheses are paired bases and dots are unpaired bases) of the shapes differ. For example, two structures that differ by exactly one base pair would have a Hamming distance of two. By setting fitness equal to a hyperbolic function of the distance to the target shape, we model strong selection for that target, that is, only molecules very similar to the target are expected to function well.

The overall fitness, *W,* of a genotype is the average of the selective values of the shapes in its ensemble of secondary structures, each weighted by its Boltzmann probability (*p_σ_*), 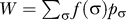 [22,24]. This fitness function assumes that both the structure of the molecule and its thermodynamic stability are important for function. The range of fitness values possible given our choice of parameters is 0.99–100.0. Prior studies show that the evolutionary dynamics are relatively robust to the particular choice of fitness function [22,24]. In our simulations, molecules replicate at each generation at a rate proportional to their fitness.

We adapted 100 replicate populations of RNA molecules under three different genomic mutation rates (*U* = 0.01, 0.08, 0.32) and 45 populations with *U* = 0.95 (this set was constrained by computational limitations). Population size was held fixed at *N* = 1,000, which was a compromise between minimizing computational time and maximizing *N*. Mutation rates were identical for all bases in the RNA alphabet. These mutation rates spanned the range of published estimates for microorganisms including viruses and bacteria [34]. Simulations each ran for 5,000 generations except the *U* = 0.95 simulations, which were computationally limited to approximately 2,500–3,000 generations.

### Identifying Fixed Mutations

Mutations were classified as fixed if at any time during the simulation they were retained by at least 95% of the extant genotypes. For each genotype containing the mutation, we verified that the mutation was always present in the lineage leading from the initial deleterious mutation, and never lost and subsequently reacquired. The subsequent analysis considers all deleterious mutations that met this 95% criteria (henceforth referred to as the “fixation threshold”). A more stringent criterion (>95%) was impractical because of the high mutation rates under which the populations evolved.

### Expected Fixation Frequencies

Kimura derived the following probability that a unique mutation with fitness effect *s* will fix in a haploid population of effective size *N_e_*: 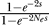 [35,36]. This model assumes that there are no subsequent changes in the mutant lineage before fixation or loss. We used Kimura's equation to predict the role of drift in the ascent of deleterious mutations and defined *N_e_* as the average number of individuals that produce progeny in each generation, which gave an upper bound for *N_e_*. Individuals produced roughly equal numbers of offspring in each generation (unpublished data), however, so the actual value of *N_e_* was likely close to this value. We emphasize that our populations significantly deviate from the idealized ones Kimura considered, and therefore these calculations are only intended to serve as a rough approximation of what might be expected to occur by drift alone. For example, under the SSWM approximation, mutations necessarily arise and proceed to fixation (or loss) one at a time; in our model, multiple mutations can simultaneously proceed to fixation or loss.

### Measuring Changes in Fitness Effect

**Figure 1:**
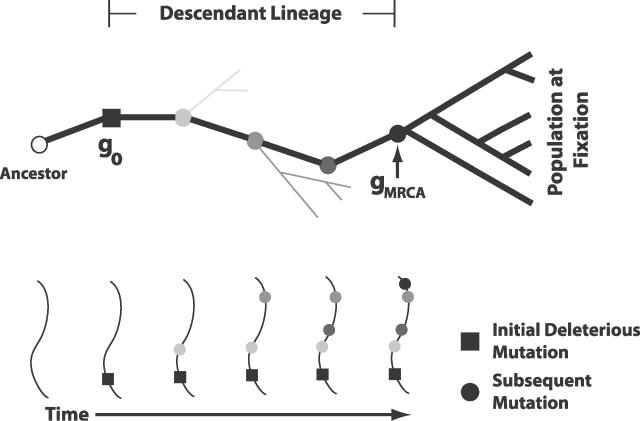
Diagram Depicting a Simplified “Descendant Lineage” for a Genotype Carrying a Deleterious Mutation (Black Square) That Fixed in the Population The genealogy of the population present at fixation is used to determine the MRCA. The descendant lineage is the single genotypic line of descent from the initial mutant genotype to *g_MRCA_*. The faint gray branches along the descendant lineage represent lineages that go extinct. The bottom half of this figure shows the accumulation of mutations in the genotypes comprising the descendant lineage.

We measured the magnitude and direction of change in the fitness effect of a deleterious mutation during its evolutionary lifetime as follows. Consider a deleterious mutation δ that creates a new mutant genotype, which we call *g_0_*. The genotype *g_0_* is the entire set of 76 bases in the molecule, including δ. If δ is not excessively severe, then *g_0_* may reproduce and its descendants possibly acquire mutation(s) at other sites. As these descendent genotypes arise, there will be a tree-like genealogy emanating from *g_0_* (Figure 1). We use *g_i_* to refer to a descendent genotype of *g_0_* containing δ and *i* subsequent mutational events at other sites (where a mutational “event” occurs during replication and creates one or more base changes). We measured the fitness effect of δ in *g_i_* by creating new genotypes in which δ was reverted back to its ancestral state, but the *i* mutational events subsequent to δ were retained. This δ-free genotype is designated 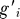 . The fitness effect of δ in the descendent genotypes *g_i_* is then 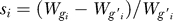 , where 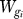 is the absolute fitness of the descendent genotype and 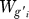 is the absolute fitness of the δ-free genotype. Informally, the fitness effect of δ is the fitness difference between the descendent genotype with and without δ.

For comparison to the fixed mutations, we selected ten deleterious mutations from each simulation (1,000 mutations for each mutation rate) and tracked the fitness effect of each mutation in the descendant genotypes, up to six subsequent mutational events. We selected these deleterious mutations at random from the subset of all deleterious mutations that met the following criteria: 1) the mutation had at least one descendant genotype, 2) the mutation did not fix, and 3) the mutation did not arise on genotypes that had one of the (eventually) fixed deleterious mutations appear within six subsequent mutations. We defined these criteria because most deleterious mutations have no descendants and therefore we cannot measure a change in fitness effect. We also modified the first criteria by increasing the required number of descendants, but this did not qualitatively change our results (unpublished data).

### Determining the MRCA of the Final Population

For each simulation, we identified the most recent common ancestor (MRCA) of the sequences present at the end of the simulation. The MRCA was exactly determined from a genotypic pedigree. It is a unique genotype; it is not, however, a consensus genotype. Typically, additional mutations arise between the origin of the MRCA and the end of the simulation that lead to divergence from the MRCA genotype. This divergence is expected given that we are evolving populations under moderately high mutation rates.

We then identified the history of mutational events on the genealogical branches leading from the founder genotype to the MRCA, thereby ignoring mutations on lineages that ultimately extinguished. We refer to the mutational events on the MRCA lineage as *ancestral* mutations. Note that these ancestral mutations may be ephemeral, never reaching substantial frequencies in the population and perhaps disappearing upon subsequent mutations at the same site occurring before the MRCA. The only requirement for an ancestral mutation is that the initial mutational event creates a genotype from which the MRCA directly descended.

## Results

### Adaptation Despite Frequent Incorporation of Deleterious Mutations

**Figure 2:**
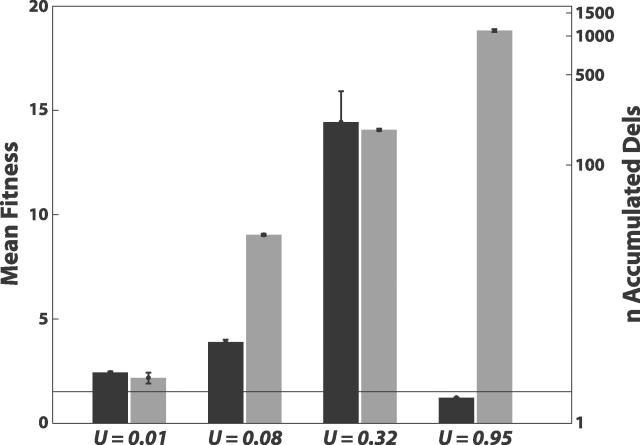
The Mean Fitness of Evolved Populations Increases with Mutation Rate (up to *U* = 0.32) despite Accumulating Greater Numbers of Deleterious Mutations The dark gray bars (left) represent the mean final fitness of populations that evolve under each mutation rate. The light gray bars (right) show the mean number of deleterious mutations that accumulate during the time to the MRCA of the final population. The error bars represent one standard error from the sample mean.

We followed the mean fitness of *n* replicate populations during 5,000 generations of evolution (*n* = 100, *U* = 0.01, *U* = 0.08, *U* = 0.32; *n* = 54, *U* = 0.95). The average fitness of the populations increased with *U* up to *U* = 0.32 and then crashed at the highest rate of *U* = 0.95 (Figure 2, dark bars). At *U* = 0.95, populations were overwhelmed by deleterious mutations and may have experienced an error catastrophe [7,8,22], though we did not investigate this possibility. In contrast to the other mutation rates, the mean final fitness achieved in the *U* = 0.32 runs was not only highest but was highly variable, with about 20% of the runs achieving extremely high fitness (>40, on a scale from 0.99 to 100.0) and the remaining runs achieving more modest fitness (≈7–9). We rejected the possibility that adaptation occasionally proceeded faster due to rare simultaneous double mutations, because such events were on average deleterious and simultaneous double mutants never fixed (unpublished data).

**Figure 3:**
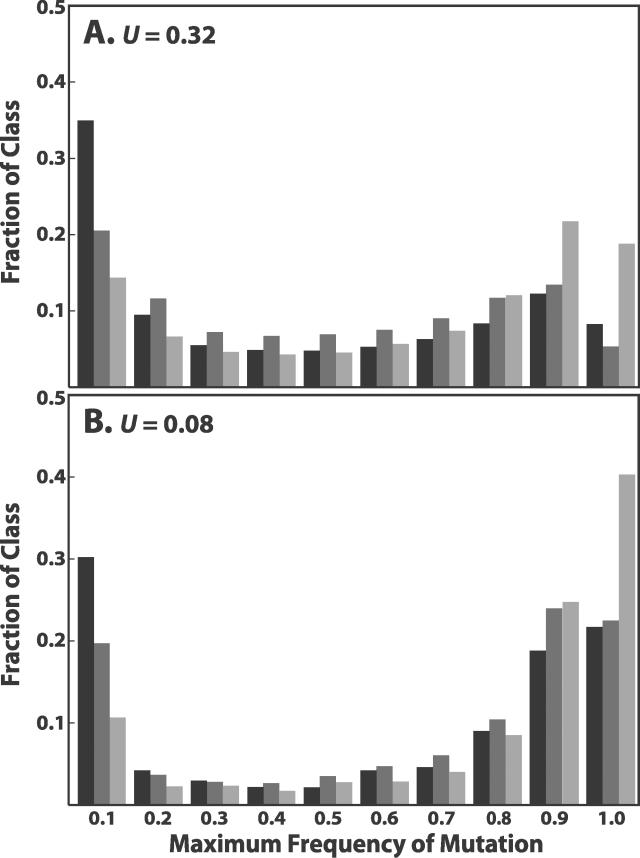
The Maximum Frequency of Ancestral Mutations (Those Forming the Dominant Mutational Lineage from the Founding Population to the MRCA of the Ending Population) That Did Not Fix The black, dark gray, and light gray bars correspond to mutations with initial fitness effects that are deleterious, neutral, and beneficial, respectively. The *x*-axis is the upper bin bound for the frequency attained (e.g., 0.20 includes those mutations whose maximum frequency is greater than 0.10 and less than or equal to 0.20). The *y*-axis is the fraction of mutations in each class that attained each frequency range. The top pane (A) shows the frequency distribution for *U* = 0.32, and the bottom pane (B) depicts *U* = 0.08.

We tallied the cumulative numbers of deleterious and beneficial *ancestral* mutations during the time leading to the MRCA of all extant sequences at the end of each simulation. Ancestral mutations are those that occur along the single dominant genotypic lineage from the founding genotype to the MRCA and define a history of sequential mutational events. A relative minority of the total ancestral mutations ultimately reached the fixation threshold—about 10% under *U* = 0.32 and 15% under *U* = 0.08 (unpublished data). Figure 3 shows the maximum frequency attained by each ancestral mutation that did not fix. Several forces may operate to preclude mutations arising on the MRCA lineage from fixing, such as drift, clonal interference, and selection for other mutations at the same site.

Each mutation in this historical sequence was classified as deleterious or beneficial according to its relative fitness effect at the time it arose. Deleterious mutations were those with a fitness effect that was less than the reciprocal of the actual population size (*s* < −1/N), while beneficial mutations were those with a fitness effect that was greater than the reciprocal of the actual population size (*s* > 1/N). Intuitively, the incidence of MRCA ancestral deleterious mutations increased with the genomic mutation rate (Figure 2, light bars). One might also expect that the rate of adaptation (change in mean fitness) would be inversely related to the rate at which deleterious mutations impact the dominant lineage. In fact, we found the opposite across all but the highest mutation rate: higher mutation rates yielded both increased accumulation of deleterious mutations and higher mean fitness (up to *U* = 0.95). In Figure 2 (dark bars), populations with *U* = 0.32 achieved higher mean fitness, on average, than those with *U* = 0.08 or *U* = 0.01, despite incorporating a greater number of deleterious mutations.

**Figure 4:**
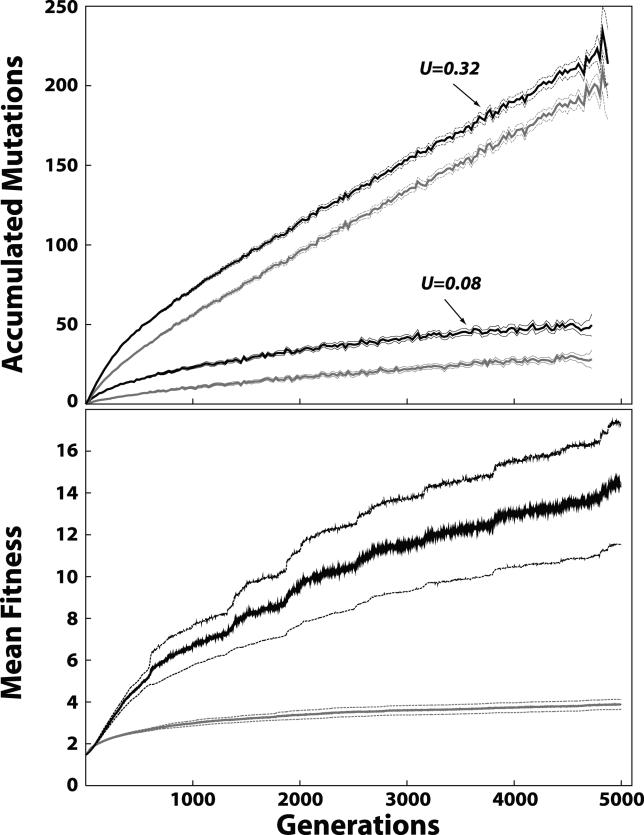
Mutation Accumulation and Mean Fitness of Evolving Populations The top pane shows the accumulation of beneficial and deleterious mutations in the lineage leading to the MRCA, while the bottom pane shows the mean fitness of the populations over time. In both panes, the thicker lines represent the mean value, and the thin, dotted lines represent bounds for 95% confidence intervals. In the top pane, the dark lines represent beneficial mutations and the light lines represent deleterious mutations (see text for definition). The jaggedness in the lines is the result of averaging over all simulations, which frequently differ in the length of their MRCA lineages. Within any single run the number of mutations monotonically increases. In the lower pane, the dark lines correspond to *U* = 0.32 and the light lines correspond to *U* = 0.08.

Figure 4 illustrates three unintuitive properties for the fitness and ancestral mutation trajectories for populations experiencing *U* = 0.08 and *U* = 0.32. First, *U* = 0.32 populations experienced substantially greater incorporation of deleterious mutations than *U* = 0.08 populations, yet enjoyed consistently higher mean fitness. Second, ancestral deleterious and beneficial mutations occurred in the MRCA lineages at nearly equal rates. The correlation between mutation rate and mean fitness may be explained, in part, by the more rapid accumulation of beneficial mutations under moderately high mutation rates. Third, during periods of relatively stable mean fitness, deleterious mutations impacted the MRCA lineage at the same rate as during periods of rapid adaptation. These observations taken together suggest that initially deleterious mutations may not strictly impede adaptation, in contrast to theoretical predictions [1–4].

### Processes Enabling the Fixation of Deleterious Mutations

We now consider the relative importance of several forces that might produce these counterintuitive observations. We focused our attention on the smaller subset of deleterious mutations that fixed in the populations. Three distinct processes accounted for the success of these mutations: 1) random genetic drift, 2) hitchhiking, and 3) fitness reversals, i.e., the fitness effect changed from bad to good.

We discuss these processes in reverse order, beginning with the most prevalent and unexpected of the three: fitness reversals driven by compensatory mutations. Suppose a deleterious mutation arises and decreases the fitness of the genotype carrying it by a factor *s*. Population-genetic models of adaptation generally assume conditions of strong selection and weak mutation, SSWM, and, therefore, during the trajectory to either fixation or loss, no additional change occurs in the genotype carrying the deleterious mutation. Under the SSWM assumptions, *s* would not be expected to change during the evolutionary trajectory of the mutation. If the SSWM assumptions are relaxed, however, a genome carrying the deleterious mutation may experience additional mutations before it fixes or is lost from the population, and thus the *s*-value of the initial mutation may change.

For each genome experiencing a deleterious mutation (*g_0_*), a complete genealogy was kept of every genotype that descended from it. A deleterious mutation (δ) was considered fixed when at least 95% of the genotypes in the extant population retained δ throughout their evolutionary histories. Starting with the extant population in which δ was first fixed, we searched backward to identify the most recent common ancestor genotype (*g_MRCA_*) of all genotypes that retained δ. Since the populations were evolved under moderately high mutation rates, *g_MRCA_* often contained δ p-lus several subsequent mutations at other sites that arose after δ and before the fixation of δ.

We identified the single-descendant lineage of genotypes that captured the history of mutations beginning at *g_0_* and ending at *g_MRCA_*: {*g_o_, g_1_,…, g_i_ ,g_MRCA_*}, which we referred to as the “descendant lineage” of δ. The typical number of subsequent mutations in the descendant lineage was 2–10 (2–40) in populations experiencing *U* = 0.08 (*U* = 0.32). Each subsequent mutation could have altered the fitness effect of δ before its fixation, and therefore we measured the fitness effect (*s_i_*) of δ at each “step” along this single descendant lineage from *g_0_* to *g_MRCA_* (δ was necessarily present at each step). We used this temporal series of *s_i_* to capture the changing fitness effect of δ.

**Figure 5:**
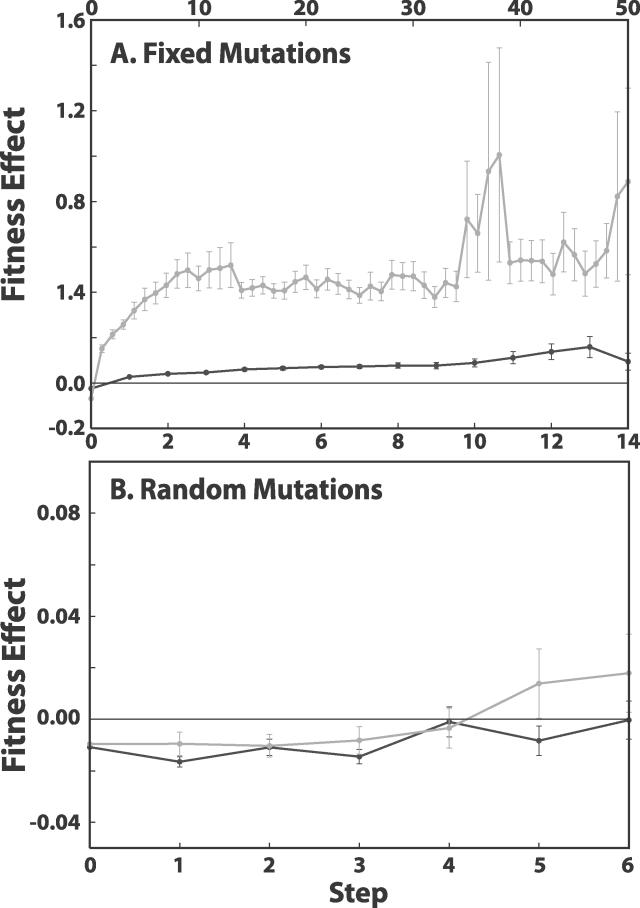
Fixed Deleterious Mutations Interact Positively with Subsequent Substitutions, while Random Deleterious Mutations Generally Remain Deleterious (A) Mutations that were initially deleterious and ultimately fixed tended to become beneficial before their fixation. It is apparent that the largest increases in fitness occurred in the first few subsequent mutations. (B) In contrast, random deleterious mutations generally remained deleterious with subsequent mutations. In both graphs, the black lines correspond to *U* = 0.08 and the light lines to *U* = 0.32 (error bars represent one standard error of the mean). The horizontal lines separate the beneficial (above) and deleterious (below) fitness effects. The fitness effect of a mutation is calculated as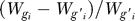 , where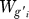 is the fitness effect of the descendent lineage without the fixed mutation and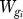 is the fitness effect of the descendent lineage *with* the fixed lineage.

Many of the deleterious mutation fixation events were characterized by dramatic fitness reversals before fixation occurred, as the genotypes containing δ accumulated additional mutations. These subsequent mutations rapidly transformed δ from a liability into an asset and, thereby, increased its probability of success (Figure 5A). Both the rate and magnitude of the fitness-effect reversals appeared to increase with mutation rate. For a random sample of deleterious mutations that never fixed, the pattern was markedly different. These mutations typically remained a liability upon subsequent mutation (Figure 5B), though the few deleterious mutations that persisted for five or six steps appeared to have acquired some small-effect compensatory mutations. Thus, most deleterious mutations remained deleterious throughout their evolutionary lifetime; only a notable few became beneficial through positive interactions with their changing genetic backgrounds. Even at *U* = 0.01, some fitness reversals were observed (unpublished data), indicating that a much lower mutation rate is required to meet the SSWM assumptions of population-genetic models.

**Figure 6:**
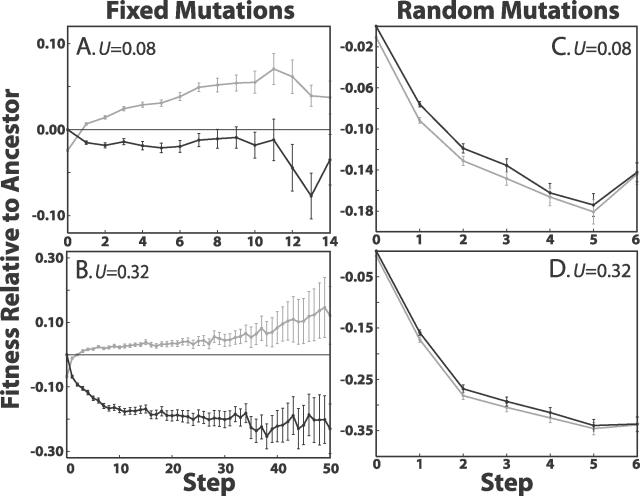
Fixed Deleterious Mutations Are Found in Genotypes That Are More Fit Than the Ancestral Genotype in Which the Deleterious Mutation Arose In each graph, the gray lines correspond to *g_i_* and the black lines to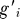 . We define the fitness of descendent genotypes relative to the ancestor as ((*W_desc_ − W_anc_)/W_anc_*), where *W_anc_* is the fitness of the parent genotype that gave rise to the deleterious mutation and *W_desc_* may mean the fitness of *g_i_* or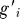 . The error bars depict one standard error of the mean. (A) and (B) show the change in fitness of the genetic backgrounds harboring the fixed deleterious mutations in the *U* = 0.08 and *U* = 0.32 runs, respectively. (C) and (D) show the change in fitness of the genetic backgrounds of random deleterious mutations that did not fix in the *U* = 0.08 and *U* = 0.32 runs, respectively.

A fitness-effect reversal does not imply that the fitness of the genotype as a whole will rise from below the ancestor to above the ancestor, or, furthermore, that the reversal explains the ultimate fixation of the initial mutation. It merely means that a genotype is better off with the mutation than without it. In Figure 6A and 6B, we show that, indeed, the fitnesses of the genotypes containing the fixed deleterious mutations (*g_i_,* light lines) rose to levels above that of the ancestor, and that without the initial mutation (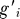 , dark lines), the fitnesses of the genotypes were significantly lower. Notably, under both the high and low mutation rates, the average fitness of the 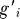 remained below that of the ancestor. For the randomly chosen deleterious mutations that did not fix (Figure 6C and 6D), the fitness of the descendant genotypes (with and without the initial mutation) continually declined relative to that of the ancestor and the fitness of the 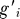 is greater than that of the *g_i_*. These figures indicate that fitness reversals via interactions with compensatory mutations played an important role in the ascent of these deleterious mutations. In fact, we found that about 80% of the initially deleterious mutations that fixed did so as a result of a fitness-effect reversal (Table 1)—a process not considered in most population genetics theory, with a few notable exceptions [18,37]. For comparison, ancestral mutations (those in the MRCA lineage) that did not fix in the population only reversed their fitness effect about 25% of the time (unpublished data).

**Table 1:**
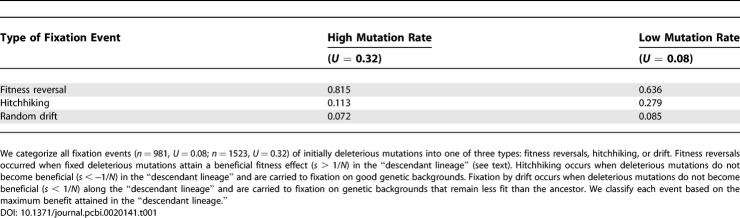
Forces Leading to the Fixation of Deleterious Mutations

We next consider the second process contributing to fixation of deleterious mutations: evolutionary hitchhiking. We say that a deleterious mutation hitchhikes to fixation when it fixes on a genetic background that attains fitness at or above the ancestor (*g_0_*), but the fixed deleterious mutation remains deleterious (or neutral) in every genotype leading to *g_MRCA_*. We determined the number of fixed deleterious mutations that did not undergo a fitness-effect reversal and existed on genotypes that evolved to higher fitness than the ancestor before the deleterious mutation fixed. Finally, we assumed that the remaining fixation events were the result of random genetic drift. These were the fixed deleterious mutations that maintained a negative (or neutral) fitness effect and were found on genotypes with fitness below the ancestor (Table 1).

We finally ask whether the number of fixation events that we attribute to fitness-effect reversals and hitchhiking might be within the range predicted to occur by drift alone. Populations experiencing genomic mutation rates of *U* = 0.08 and *U* = 0.32 fixed, on average, 9.8 and 15.2 initially deleterious mutations, during 5,000 generations of evolution, respectively. This corresponds to actual fixation rates for deleterious mutations of 3.09 × 10^−4^ (95% CI: 2.90 × 10^−4^, 3.29 × 10^−4^) and 1.11 × 10^−4^ (95% CI: 1.05 × 10^−4^, 1.15 × 10^−4^), for *U* = 0.08 and *U* = 0.32, respectively (Table 2). Kimura's approximation yields expected fixation probabilities of 3.27 × 10^−5^ (*U* = 0.08) and 1.21 × 10^−31^ (*U* = 0.32) when calculated using the mean fitness effect of the fixed deleterious mutations in our simulations [*s* = −0.0239 (*U* = 0.08); *s* = −0.0672 (*U* = 0.32)]. A comparison of the observed and expected rates of fixation suggests that, under *U* = 0.32, fitness reversals may lead to rates of deleterious mutation fixation that are higher than expected by drift alone, while under *U* = 0.08 the rates of deleterious fixation do not exceed the expected rates from Kimura's model. We stress, however, that our populations are significantly different from the idealized ones Kimura envisioned and thus there may be multiple reasons for the observed discrepancies.

**Table 2:**
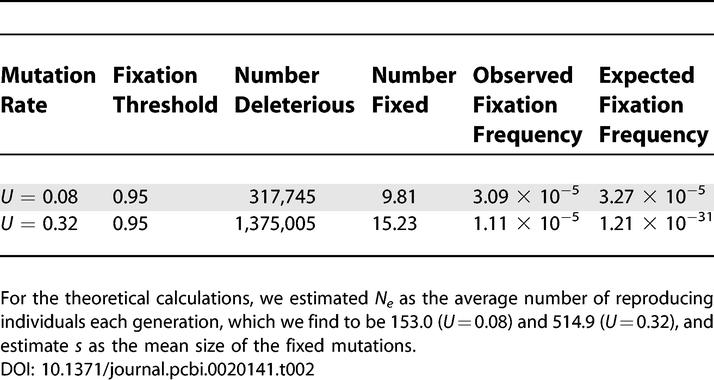
The Probability of Fixation of Deleterious Mutations in Our Simulations Compared with Theoretical Predictions of Kimura (1957)

In summary, a complicated mix of forces allowed initially deleterious mutations to occasionally rise to fixation. Hitchhiking may work in concert with fitness-effect reversals, and therefore our estimates of the contributions of these two processes may be low (Table 1). Furthermore, the incidence of fitness reversals increased with mutation rate (from *U* = 0.01 to *U* = 0.08 to *U* = 0.32), perhaps contributing to the evolution of higher mean fitnesses across these mutation rates. Fitness-effect reversals are only part of the story, however, as the initial deleterious effects of fixed deleterious mutations were much larger in the *U* = 0.32 populations than in the *U* = 0.08 populations.

## Discussion

In this study, we offer a new perspective on the effect and role of deleterious mutations in adaptation. We simulated the adaptation of asexual populations of 1,000 individual RNA genomes that each coded for a phenotype, which consisted of a set of thermodynamically probable secondary structures. In turn, fitness depended on the overall similarity of a molecule's phenotype to a target shape. The effect of a mutation was determined by measuring its impact on the shape of the molecule (its phenotype), and thus the distribution of fitness effects behaved as might be expected of a biological system.

The novel result is that nearly one-third of the mutations that evolve in the MRCA lineage (the single genealogical history from the starting genotype to the MRCA of the ending population) arise with deleterious effects, yet this apparent load of deleterious mutations does not impede adaptation. This can be explained by the frequent occurrence of fitness reversals, that is, more than half of these deleterious mutations do not stay deleterious, but become neutral or beneficial through interactions with compensatory mutations. Importantly, the compensatory mutation(s) arise and reverse the deleterious effect well before the deleterious mutation fixes, and the beneficial combination of mutations then ascends together to fixation.

Kimura described a special case of our process in a model of neutral compensatory mutations [18]. He derived the time for transition between a wild-type genotype (AB) to a double mutant (A′B′), which has fitness identical to the wild-type. To create the double mutant, however, the population needed to pass through the deleterious intermediates A′B or AB′, which each had fitness 1 − *s*. He showed that under continuous mutation pressure, the double mutant can fix relatively rapidly, even in large populations. The fixation time for the double mutant was not unreasonably long, being slightly longer than the fixation time for a pair of neutral mutations and much shorter than the fixation time for a pair of unconditionally deleterious mutations.

A major question is whether this process occurs in nature. There is abundant experimental evidence that the fitness effect of a mutation can depend on genetic background [37–39]. There is also evidence that compensated deleterious mutations are present in the genomes of flies [40] and humans [41]. There is, however, a lack of empirical evidence (as opposed to negative evidence) for the full process we describe, although it would be difficult to observe without detailed histories of the substitution events. In a study using the AVIDA software, Lenski and colleagues observed a moderate number (15%) of initially deleterious mutations that ultimately fixed. One of those deleterious mutations reversed its fitness effect and provided the basis for further fitness gains, though it was not stated whether the reversal occurred before fixation [42].

Two factors may be necessary for this process to occur: a high mutation rate and epistasis. The mutation rate must be high enough that a second, interacting mutation arises in the genome before the first mutation is lost or fixed. While background selection typically refers to pairs of mutations (one beneficial and one deleterious) that have net negative fitness effects and no epistatic interactions, here we focus on pairs of mutations (with at least one deleterious) that epistatically interact to yield net positive fitness effects. We conjecture that, to the extent background selection is occurring, fitness reversals may likewise be important to the evolutionary dynamics. Furthermore, the mutation rate of interacting sites must be high enough to have a reasonable probability of creating the right combinations. Some natural systems are characterized by high mutation rates, including RNA viruses. Additionally, there is a sense that the self-replicating molecules present at the origin of life may have had high error rates, and so may fit this model. In the early stages of the process, a small population size may be important to the extent that it affects the rate of drift.

Ascent via fitness reversals also requires a rugged (epistatic) fitness landscape. Although epistasis is widely recognized in genetics and evolution, the process described here requires an extreme form of it: the fitness effect of a mutation actually reverses (from bad to good) in the presence of a second mutation. Most studies of epistasis focus on the weaker form in which the fitness effect of a first mutation undergoes small changes in response to a second interacting mutation.

Recent theoretical and experimental efforts, however, are beginning to elucidate additional details of these stronger epistatic interactions (so-called “sign epistasis”) [37,39]. For instance, one recent study of cefotaxime resistance demonstrated strong epistatic interactions between mutations [39]. Their findings, however, were interpreted within the same SSWM assumptions previously mentioned. As a result, they reached the conclusion that the evolutionary optimization process is limited to a succession of individual mutations that each increase fitness. If the SSWM assumptions are relaxed, however, then many more evolutionary trajectories may be possible, in particular those that involve deleterious mutations followed by compensatory mutations that reverse the initial deleterious effect.

Other studies suggest that compensatory mutations occur at relatively high frequencies [15,43]. For example, in the virus φX174, Poon and Chao estimated that fitness recovery following a deleterious mutation proceeded by compensatory mutation (as opposed to back mutation) in about 70% of the cases examined [15]. As another example, Poon et al. estimated, using data from 129 deleterious mutations in a wide range of organisms and genes, that approximately 12 compensatory mutations exist for each deleterious mutation [43]. Compensatory evolution, as we observed in simulated RNA, may therefore be a general feature of more complex organisms.

Our results are a natural extension of previous work examining compensatory evolution in viruses and bacteria. As noted above, those studies almost exclusively considered compensatory beneficial mutations appearing after the fixation of a deleterious mutation and demonstrated that the compensatory effect depends on the presence of the initial deleterious mutation [13–15,44]. The compensatory interactions we observe occur *prior* to fixation (or loss) of the deleterious mutation, and thus have a fundamentally different evolutionary implication: they alter the fitness effect of a deleterious mutation sufficiently early to sway its ultimate evolutionary fate.

While it is widely recognized that asexuality poses several problems to adaptation through processes such as clonal interference, background selection, and Muller's ratchet [45], the relative contributions of each to the “cost of asexuality” is not known. A natural extension of this study is, therefore, to partition the fates of beneficial and deleterious mutations into this broader set of mechanisms. Classifying just the processes preventing fixation of beneficial mutations, however, would be nontrivial. In our model, all processes that affect the fates of beneficial mutations are occurring simultaneously, and, furthermore, epistasis is rampant and a mutation will typically be followed by others before fixation or loss.

In our study, deleterious mutations accumulated rapidly without impeding adaptation—a result counter to most theoretical predictions. We attribute our results, at least in part, to the fact that the fitness effect of a mutation can change dramatically and rapidly upon additional mutations. It remains unclear whether these reversions are sufficient not only to ensure fixation of the original mutation, but also to constitute major adaptive steps.

## Abbreviations

MRCA: most recent common ancestor
SSWM: strong selection, weak mutation
